# Translocation of LRP1 targeted carbon nanotubes of different diameters across the blood–brain barrier *in vitro* and *in vivo*

**DOI:** 10.1016/j.jconrel.2016.01.031

**Published:** 2016-03-10

**Authors:** Houmam Kafa, Julie Tzu-Wen Wang, Noelia Rubio, Rebecca Klippstein, Pedro M. Costa, Hatem A.F.M. Hassan, Jane K. Sosabowski, Sukhvinder S. Bansal, Jane E. Preston, N. Joan Abbott, Khuloud T. Al-Jamal

**Affiliations:** a Institute of Pharmaceutical Science, Faculty of Life Sciences & Medicine, King's College London, Franklin-Wilkins Building, 150 Stamford Street, London SE1 9NH, UK; b Centre for Molecular Oncology, Barts Cancer Institute, Queen Mary University of London, London EC1M 6BQ, UK

**Keywords:** Nanomedicine, Targeting, Transcytosis, Angiopep-2, Brain delivery, Carbon nanotube

## Abstract

Brain glioblastoma and neurodegenerative diseases are still largely untreated due to the inability of most drugs to cross the blood–brain barrier (BBB). Nanoparticles have emerged as promising tools for drug delivery applications to the brain; in particular carbon nanotubes (CNTs) that have shown an intrinsic ability to cross the BBB *in vitro* and *in vivo*. Angiopep-2 (ANG), a ligand for the low-density lipoprotein receptor-related protein-1 (LRP1), has also shown promising results as a targeting ligand for brain delivery using nanoparticles (NPs). Here, we investigate the ability of ANG-targeted chemically-functionalised multi-walled carbon nanotubes (*f*-MWNTs) to cross the BBB *in vitro* and *in vivo*. ANG was conjugated to wide and thin *f*-MWNTs creating *w*-MWNT-ANG and *t*-MWNT-ANG, respectively. All *f*-MWNTs were radiolabelled to facilitate quantitative analyses by γ-scintigraphy. ANG conjugation to *f*-MWNTs enhanced BBB transport of *w*- and *t*-MWNTs-ANG compared to their non-targeted equivalents using an *in vitro* co-cultured BBB model consisting of primary porcine brain endothelial cells (PBEC) and primary rat astrocytes. Additionally, following intravenous administration *w*-MWNTs-ANG showed significantly higher whole brain uptake than the non-targeted *w*-MWNT *in vivo* reaching ~ 2% injected dose per g of brain (%ID/g) within the first hour post-injection. Furthermore, using a syngeneic glioma model, *w*-MWNT-ANG showed enhanced uptake in glioma brain compared to normal brain at 24 h post-injection. *t*-MWNTs-ANG, on the other hand, showed higher brain accumulation than *w*-MWNTs. However, no significant differences were observed between *t*-MWNT and *t*-MWNT-ANG indicating the importance of *f*-MWNTs diameter towards their brain accumulation. The inherent brain accumulation ability of *f*-MWNTs coupled with improved brain-targeting by ANG favours the future clinical applications of *f*-MWNT-ANG to deliver active therapeutics for brain glioma therapy.

## Graphical abstract

**Figure:**
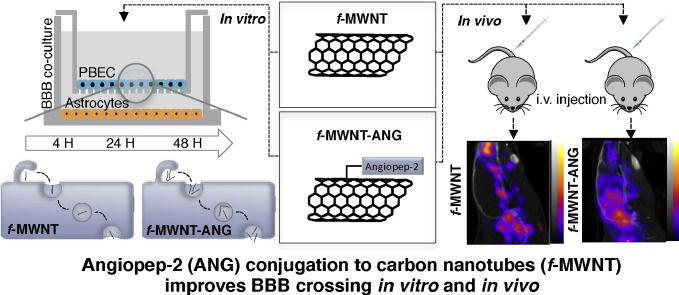

## 1 Introduction

The blood–brain barrier (BBB) is a major obstacle for drug delivery to the brain, limiting the number of drugs reaching the market to tackle brain disorders [1]. Nanoparticles (NPs), providing a flexible platform for conjugating drugs and targeting ligands, have been extensively researched to facilitate BBB crossing and effective delivery to the brain [2].

Carbon nanotubes (CNTs) have several attractive characteristics in this regard, including a high aspect ratio, and the ability to penetrate biological membranes due to their tubular shape [3]. *f*-MWNTs injected directly into the brain led to a significant reduction in neuronal damage in rats [4], and also led to improved motor function recovery in an Endothelin-1 rat stroke model *via* the successful delivery of caspase-3 siRNA to neurons in mouse brain cortex [5]. *f*-MWNTs also improved the delivery of CpG oligonucleotide into glioma tumour cells using a single intracranial injection [6]. In spite of the encouraging results, none of the above-mentioned studies utilised the systemic route of administration.

Recently we demonstrated the ability of *f*-MWNTs to cross the BBB *in vitro* and *in vivo* [7]. The *in vitro* BBB model consists of primary porcine brain endothelial cells (PBEC) co-cultured with rat astrocytes. *f*-MWNTs were visualised within endocytic vesicles of PBECs and crossed the monolayer within 24 h. Following intravenous (i.v.) administration, radiolabelled *f*-MWNT was also found to reach mouse brain without the use of a targeting ligand.

The current study further explored *f*-MWNT as a brain delivery nanocarrier by introducing a brain-targeting ligand Angiopep-2 (ANG) to its surface [8]. ANG is a 2.4 kDa peptide of the Angiopep peptide family which has a high affinity for the low-density lipoprotein receptor-related protein-1 (LRP1). LRP1 is a multifunctional receptor, expressed in several tissues including brain capillary endothelium and gliomas [9], [10]. ANG exhibited significantly greater transcytosis capacity across bovine brain endothelial cells compared to transferrin, lactoferrin and avidin [11]. Studies have shown that the direct conjugation of ANG to drugs such as paclitaxel and neurotensin enhanced brain delivery, and showed significant tumour reduction and analgesic effects, respectively [8], [12]. ANG conjugation to NPs also showed significant improvement in brain delivery of polymeric micelles [13], dendrimers [14] and gold nanoparticles [15]. Based on the reported success in previous studies, we hypothesised that conjugating ANG to *f*-MWNT would improve its BBB permeation and also facilitate brain tumour targeting *in vitro* and *in vivo*.

*f*-MWNTs of two different diameters were employed in this study: wide MWNTs (*w*-MWNTs, diameter 24.5 ± 1.7 nm, the *f*-MWNT used in our previous study [7]) and thin MWNTs (*t*-MWNTs, diameter 7.8 ± 1.5 nm). The transport of non-targeted (*w*- and *t*-MWNT) and targeted (*w*- and *t*-MWNT-ANG) *f*-CNTs was studied firstly using the *in vitro* BBB co-culture model as previously reported [7]. *In vivo* brain uptake of radiolabelled *f*-MWNTs was then studied by quantitative γ-scintigraphy, 3D-single photon emission computed tomography/computed tomography (SPECT/CT) imaging and autoradiography following i.v. administration in normal and glioma-bearing mice.

## 2 Materials and methods

### 2.1 Materials

Pristine MWNTs, used for preparation of *w*-MWNTs (diameter 20–30 nm; length 0.5–2.0 μm, batch #1237YJS) were purchased from Nanostructured and Amorphous Materials Inc., USA. Pristine MWNTs used for the preparation of *t*-MWNTs (diameter 9.5 nm; length 1.5 μm; batch #NC3100) were purchased from Nanocyl s.a., Belgium. Reagents for chemical functionalisation of *f*-MWNT, PBEC and astrocyte isolation and culturing are listed in SI. Costar Transwell™ permeable 3.0 μm pore polycarbonate supports were purchased from Corning Inc., USA. Chemicals for the Angiopep-2 synthesis, fluoropore PTFE hydrophobic 0.22 μm membrane and glutaraldehyde 25% aqueous solution were purchased from Merck Millipore, UK. The radioactive tracer [^111^In]Cl_3_ was obtained from Mallinckrodt Pharmaceuticals (Netherlands) as an aqueous solution in 0.5 M HCl and used without further purification. Carbon/formvar coated 300-mesh copper grids were obtained from Agar Scientific, UK.

### 2.2 ANG synthesis by solid phase peptide synthesis (SPPS)

The synthesis of a modified ANG sequence (TFFYGGSRGKRNNFKTEEYG) [8] was carried out using the CEM Liberty 1 microwave peptide synthesiser (CEM Microwave Technology Ltd., UK) on NovaSyn® TGR resin (0.5 mmol/g loading) as previously described [16]. Further details on SPPS are provided in SI. The molecular weight of the synthetic ANG was confirmed by matrix-assisted laser desorption/ionisation time of flight mass spectrometry (MALDI-TOF MS) (Bruker, USA) using α-cyano-4-hydroxycinnamic acid as the matrix. ANG was also characterised by reverse phase high-performance liquid chromatography (RP-HPLC) using Vydac 218TP54 column (C8, 5 μm, 4.6 × 250 mm) (Phenomenex, UK) with a water/acetonitrile gradient mobile phase containing 0.1% TFA. The eluent was detected using a UV–vis detector at 280 nm.

### 2.3 Preparation of *f*-MWNT and conjugation with ANG

**Scheme 1:**
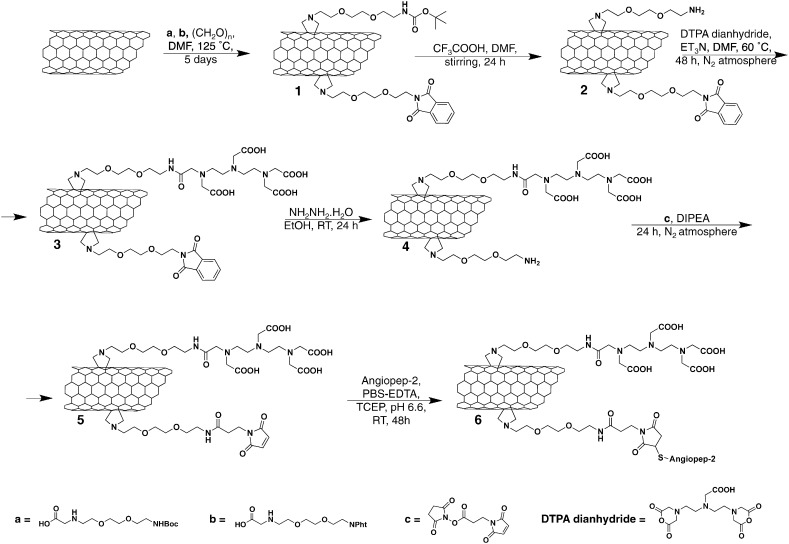
Synthesis of *w*-MWNTs.

**Scheme 2:**
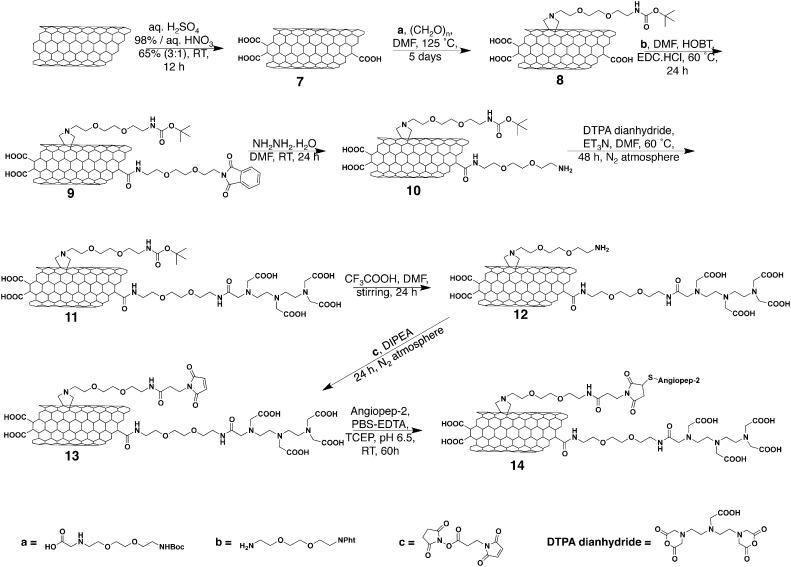
Synthesis of *t*-MWNTs.

MWNT constructs were functionalised as described previously [7], [17]. Synthesis procedures are detailed in the **SI Text** and summarised in Scheme 1, Scheme 2. The synthesis of Boc- and phthalimide-protected amino acids has been previously reported [18], [19]. To conjugate ANG to the surface of *w*- and *t*-MWNT-maleimide, 5 mg of MWNT **5** and **13** were dispersed in PBS (4 mM EDTA, 10 mM TCEP, pH 6.5) and sonicated for 10 min. ANG solution (3.4 μmol, 8.5 mg for MWNT **5** and 1.5 μmol, 3.7 mg for MWNT **13**) was mixed with the MWNT **5** and **13**, and the reaction mixture was stirred for 60 h at room temperature. Multiple centrifugation steps were carried out (15 min, 2916*g*) in water to remove the unreacted peptide from the solution. All conjugates were characterised by thermogravimetric analysis (TGA) and Transmission Electron Microscopy (TEM) as described in SI.

### 2.4 Radiolabelling of *f*-MWNT derivatives

*f*-MWNTs were radiolabelled with indium-111 (^111^In) as described previously [7], [17]. In brief, *f*-MWNT solutions (1 mg/ml in water) were mixed with ammonium acetate buffer, yielding a final acetate buffer of 0.2 M, pH 5.5, to which 10 MBq of ^111^InCl_3_ was added. The reaction was carried out for 30 min at room temperature, and terminated by the addition of EDTA quenching solution (0.1 M, 1:20 *v*/v) to chelate the unreacted ^111^In. The labelling efficiency was determined by thin layer chromatography (TLC). TLC was carried out in 0.1 M ammonium acetate mobile phase containing 50 mM EDTA, and allowed to dry before counting the signals using the Cyclone phosphor detector (Packard Biosciences, UK). The spots at the application point of the TLC strips indicated the presence of labelled *f*-MWNTs whereas the signals at the solvent front were indicative of [^111^In]EDTA. Any unbound ^111^In was removed by a centrifugation step (18,363*g*, 30 min). The stability of the labelled materials was then tested by mixing with an equal volume of serum and incubating at 37 **°**C for 24 h after labelling. Centrifugation was performed to pellet *f*-MWNTs, which were re-suspended in sterile PBS (1 mg/ml) to remove unbound ^111^In prior to *in vitro* and *in vivo* studies.

### 2.5 Setting up the BBB co-culture Transwell™ system

A co-culture BBB model comprising PBECs and primary rat astrocytes was established in our previous study to assess the transport of *f*-MWNTs *in vitro* [7]. The PBEC isolation method was originally developed by Rubin et al. [20], and further modified and improved by Abbott and co-workers [21]. Primary astrocytes type I were isolated from 0 to 2 day old Wistar rat pups following the method of Abbott et al. [22]. Briefly, astrocytes were cultured into a 12-well plate coated with 10 μg/ml poly-*L*-lysine one week before the experiment. PBECs were cultured in T-75 flasks initially and sub-cultured onto 3.0 μm pore polycarbonate inserts (Transwell™ filters) when the cells reached 50% confluence. The Transwell™ filters containing PBECs were placed in the wells above the astrocytes. The cells were incubated at 37 °C in a humidified chamber with 5% CO_2._ The trans-endothelial electrical resistance (TEER) was measured (EVOM epithelial voltohmmeter with STX2 electrode, World Precision Instruments, UK) to assess the tightness of the BBB model. *In vitro f*-MWNTs transport studies were performed when TEER values were > 200 Ω·cm^2^.

### 2.6 Transport studies of *f*-MWNTs across the *in vitro* BBB model

Serum-free medium supplemented with tight junction growth factors was replaced for 24 h before starting uptake studies to stimulate the formation of tight junctions between adjacent endothelial cells [21]. ^111^In-labelled *f*-MWNTs (20 μg/ml) were added to the apical chamber of the Transwell inserts, and the plate was incubated at 37 °C up to 72 h. At specified time points (1, 3, 5, 10, 15, 30, 45, 60, 120, 240, 1080, 2880 and 4320 min), 0.5 ml aliquots were taken from the basal chamber and stored in counting vials. The medium in the basal chamber was replenished with warm medium to maintain sink conditions. ^111^In signals were quantified as counts per minute (CPM) using a 1282 CompuGamma CS Gamma Counter (LKB Wallac, USA). Percentage uptake was calculated from the total dose added to each well. Results were expressed as mean ± S.D. (*n* = *3*). The apparent permeability coefficient (P_app_) was calculated using the following equation [23];

$$P_{\mathrm{app}}=\frac{\Delta C_{R\;}*V_{R}}{\mathrm{\Delta t}*A*C_{0.}}$$

where *ΔC*_*R*_/*Δt* is the change in concentration in the receiving chamber over time. This value was taken as the slope of the linear correlation (concentration *vs* time) from short time point intervals (3 min–60 min). *C*_*0*_ is the starting concentration in the donor chamber (CPM), *V*_*R*_ is the volume of the donor chamber (ml) and *A* is the surface area of the Transwell™ filter (cm^2^).

### 2.7 Glioma cell culture

The GL261-Luc murine glioma cells (Caliper Life Sciences, UK) were cultured in Advanced RPMI media supplemented with 10% FBS, 50 U m/l penicillin, 50 μg m/l streptomycin, 1% l-glutamine, at 37 °C in 5% CO_2_. Cells were routinely grown in 75 cm^2^ tissue culture flasks and passaged twice a week at 80% confluency using Trypsin/EDTA.

### 2.8 Organ biodistribution of *f*-MWNTs in mice by γ-scintigraphy

All *in vivo* experiments were conducted under the authority of Project and Personal Licences granted by the UK Home Office and the UKCCCR Guidelines (1998). Female C57/Bl6 mice aged 5–6 weeks were anaesthetised by isoflurane inhalation and intravenously (i.v.) injected with radiolabelled *f*-MWNTs (50 μg, 0.5 MBq) *via* a tail-vein injection. The blood was sampled at 2 min, 5 min, 30 min, 1 h, 4 h and 24 h after injection, and whole-body perfusion was performed on animals using 25 ml of heparinised saline (1000 U/L) through the left ventricle of the heart to wash out residual or loosely-bound *f*-MWNTs from the blood vessel*s.* Major organs including skin, liver, spleen, kidneys, heart, lungs, muscle, bone, brain, stomach and intestine were then harvested at 5 min, 30 min, 1 h, 4 h and 24 h post-injection. For the collection of urine and faeces, the animals grouped for the 24-h time point were caged individually in metabolic cages. Animals were starved but with access to water for 24 h while urine and faeces were collected. Excised organs were weighed and the radioactivity was measured by γ-scintigraphy (LKB Wallac 1282 CompuGamma, PerkinElmer). The results are expressed as % injected dose per organ (%ID, mean ± S.D., *n* = *3*) or % injected dose per gram tissue (%ID/g, mean ± S.D., *n* = *3*) and were statistically analysed using one-way ANOVA.

### 2.9 Brain capillary depletion

Brain tissues were subjected to a further capillary depletion method to separate the parenchyma from brain capillaries following a previously published method [24]. Each brain was placed in a glass homogeniser in 0.8 ml of ice-cold depletion buffer (10 mM HEPES in HBSS, pH 7.4). The brain was homogenised with 15 stokes of the pestle, and 1.6 ml of depletion buffer, 26% dextran (148 kDa), was added into the homogeniser. The brain was further homogenised with 3 strokes of the pestle. Brain homogenate was centrifuged at 3220*g* for 15 min. Brain parenchyma (supernatant) and brain vasculature (pellet) fractions were separated and the radioactivity was measured using gamma counting. The results are expressed as %ID per total brain capillaries or total brain parenchyma (mean ± S.D., *n* = *3*) and statistically analysed using one-way ANOVA. The area under the curve (AUC) was calculated following the trapezoidal method by fitting the data set to a linear and log-linear model after 1 and 24 h, respectively [25]. The elimination rate constant was extrapolated from plotting the changes in concentrations versus time.

### 2.10 SPECT/CT imaging

*In vivo* brain uptake of *f*-MWNTs was also examined by live SPECT/CT imaging. Female C57/Bl6 mice aged 5–6 weeks were anaesthetised by isoflurane inhalation and injected with *f*-MWNTs (50 μg, 7–10 MBq) *via* a tail vein. SPECT/CT imaging was carried out at 0–30 min, 4 h and 24 h post-injection in the prone position using the NanoSPECT/CT scanner (Bioscan, USA). SPECT scanning was acquired over 24 projections (60 s per projection) using a 4-head scanner with 1.4 mm pinhole collimators for a total acquisition time of 30–40 min. CT scans were performed using a 45 kVP X-ray source after each SPECT. SPECT images were reconstructed using HiSPECT (Scivis GmbH, Germany) and CT images were reconstructed using InVivoScope, proprietary Bioscan software. The two images were fused and analysed by InVivoScope.

### 2.11 Autoradiography

To investigate the regional distribution of *f*-MWNT in brain, mice were i.v. injected with *f*-MWNTs (50 μg CNT in 100 μl PBS, ~ 7 MBq), and the brains were isolated at 5 min after injection for autoradiography. Each brain was cut into 2 mm thick coronal sections using a mouse brain slicer (Zivic-Miller, USA). Sections were placed between two glass microscope slides, in contact with a super-sensitive plate (Cyclone® storage phosphor screen, Packard) for ~ 17 h in autoradiography cassettes (Kodak BIomax Cassette®, Carestream Health Inc., UK), which were then scanned using cyclone phosphor detector (Packard Biosciences. UK).

### 2.12 Brain uptake of *f*-MWNTs in glioma-bearing mice

C57/Bl6 mice bearing intracranial GL261 glioma were prepared to assess the brain uptake of *f*-MWNTs in a syngeneic glioma model. Female C57/Bl6 mice aged 6–8 weeks were anesthetised by isoflurane inhalation and injected stereotactically with 1.25 × 10^5^ GL261-Luc glioma cells (3 μl in PBS) into the right hemisphere using a Hamilton syringe (Harvard Apparatus, UK) with a 26-gauge needle at 0.2 μl/min. The stereotactic coordinates relative to bregma were: 3 mm anterior, 1 mm lateral, 3 mm deep. Following surgery, *in vivo* quantitative bioluminescence imaging was performed twice a week to monitor the tumour growth (IVIS Lumina III, Perkin-Elmer, UK). Mice under anaesthesia were subcutaneously injected with luciferin (D-luciferin potassium salt, Perkin-Elmer, UK) at 150 mg/kg and imaged 8 min after injection. Bioluminescence signals from regions of interest were measured using Living Image software (Perkin-Elmer, UK) and recorded as total flux (photons/s). Mice were i.v. injected with radiolabelled *f*-MWNTs when tumours reached desired size roughly 2–3 weeks after tumour implantation (total flux > 1 × 10^6^ p/s). At 24 h post*-*injection, mice were perfused as described previously and brains were isolated and measured by γ-scintigraphy.

## 3 Results

### 3.1 Characterisation of ANG peptide

A modified ANG peptide (TFFYGGSRGKRNNFKTEEYG) [8] was synthesised using microwave-assisted SPPS and characterised with HPLC and mass spectrometry. Fig. S1 A shows the structure of ANG with propargylglycine (G20) conjugated to the C-terminal. The addition of cysteine facilitates the conjugation to the modified *f*-MWNTs *via* thiol–maleimide click reaction. Fig. S1 B shows the HPLC chromatogram of ANG. A single peak was observed (retention time ~ 16.7 min) confirming the purity of ANG. The synthesised ANG was characterised by MALDI-TOF mass spectrometry. The highest abundant ion in the mass spectrometry chromatogram corresponds to 2499.13 m/z (Fig. S1 B, inset), which corresponds to the predicted molecular weight of ANG.

### 3.2 Functionalisation of *w*-MWNT and *t*-MWNT with ANG

The extremely small diameter of pristine *t*-MWNTs makes them susceptible to aggregation in aqueous solution. Therefore, *t*-MWNTs were initially oxidised in a mixture of sulphuric and nitric acid to increase the water dispersibility. The acid treatment introduced carboxylic functional groups on the side-walls of *t*-MWNT allowing more efficient subsequent derivatisation with functional groups [17]. Pristine *w*- and oxidised *t*-MWNTs were functionalised using 1, 3-dipolar cycloaddition. *t*-MWNT was then subjected to amidation reaction as described previously. Details of the functionalisation steps are shown in Scheme 1, Scheme 2 as we previously reported [7], [17]. DTPA dianhydride was conjugated to the amine terminal to chelate the radioactive tracer ^111^In used to track *f*-MWNT *in vitro* and *in vivo*. The phthalimide-deprotected amines were modified with a maleimide group to couple the thiol-modified ANG *via* thiol–maleimide click reaction.

### 3.3 Characterisation of *f*-MWNTs

TGA was used to quantitatively monitor the functionalisation steps of *f*-MWNTs. TGA relies on measuring the weight loss after heating the sample to 1000 °C under N_2_ atmosphere. The weight loss is directly related to the introduction of functional groups onto the side-walls of *f*-MWNTs. The weight loss was determined at 600 °C, the temperature at which all the functional groups decompose, but below the decomposition temperature of carbon nanotubes. Fig. S2 A and B show the TGA profiles of *w*-MWNTs and *t-*MWNTs, respectively. The measured weight loss and the corresponding functional group contents are summarised in Table 1. The resulting degree of functionalisation for MWNT **6** (*w*-MWNTs-ANG) and **14** (*t*-MWNTs-ANG) were 31.2 and 7.6 μmol ANG per gramme of CNTs, respectively.

**Fig. 1:**
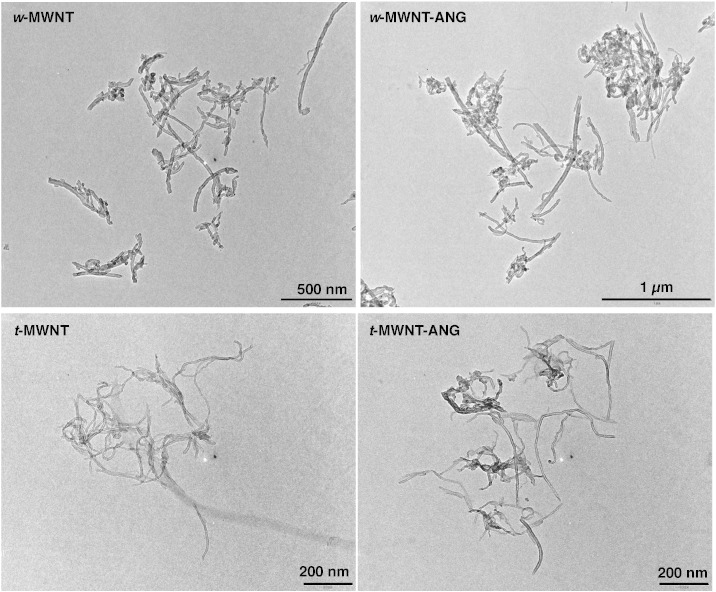
The structure of the *f*-MWNTs examined by transmission electron microscopy (TEM). *f*-MWNTs (1 mg/ml in water) were deposited on carbon/formvar coated 300-mech grid and imaged by TEM. Electron micrographs show the *w*-MWNTs (top row) and *t*-MWNTs (bottom row) before and after conjugation to ANG peptide. *f*-MWNTs showed no sign of structural damage following the functionalisation reactions and the conjugation of ANG. The diameter and length of the *w-*MWNT and *t*-MWNT were measured from TEM images using NIH Imgae J software (*n* = 50) and summarised in Table 1.

TEM imaging was used to examine the structure of *f*-MWNTs before and after coupling to ANG (Fig. 1). The images show no apparent structural differences after the coupling to ANG. The mean diameter/length of the *w*-MWNTs and *t*-MWNTs measured from TEM images were 23.8 ± 2.1/494 ± 54 nm and 7.9 ± 1.8/437 ± 61 nm, respectively (Table 1). The measured length of the *f*-MWNTs was shorter than the pristine material due to acid treatment and/or the multiple filtration steps in the functionalisation process.

### 3.4 Radiolabelling of *f*-MWNTs

*f*-MWNT constructs were radiolabelled with ^111^In and the labelling efficiency was measured by TLC (Fig. S3). The results show the labelling efficiency of ~ 21.5% and 7.0% for *w*-MWNTs and *w*-MWNTs-ANG (Fig. S3 A), while higher labelling efficiency of ~ 85.6% and 82.1% was obtained for *t*-MWNTs and *t*-MWNTs-ANG, respectively (Fig. S3 B). The free ^111^In was removed by centrifugation where the labelled *f*-MWNTs were pelleted while [^111^In]EDTA remained in the supernatant. Most labelled *f*-MWNTs remained stable (> 75%) after 24 h of incubation in 50% serum at 37 °C (Fig. S3 C).

### 3.5 ANG conjugation improves *f*-MWNTs transport across the *in vitro* BBB model

**Fig. 2:**
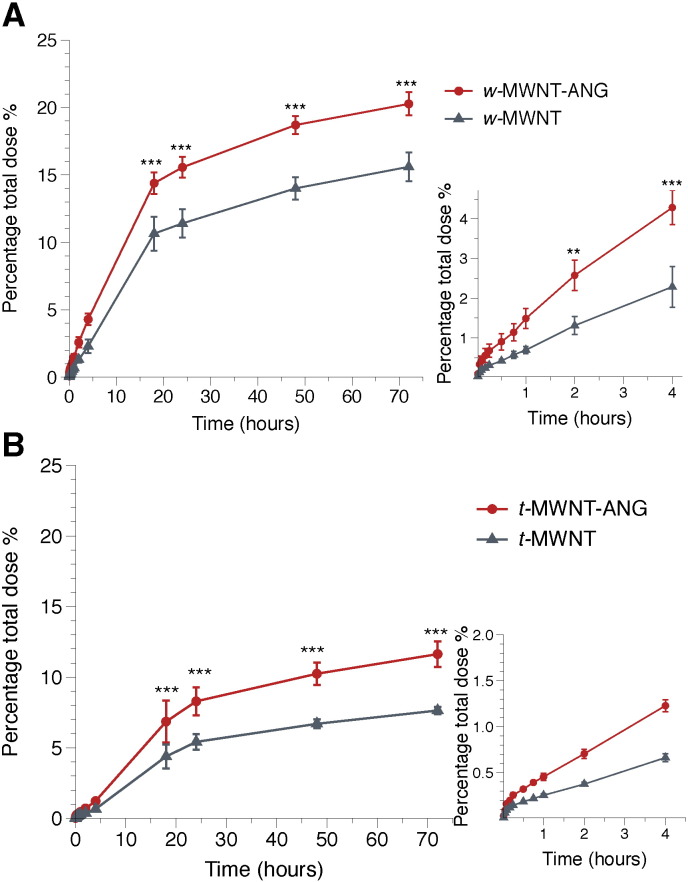
Transport of *f*-MWNTs across the *in vitro* BBB model over 72 h. Radiolabelled *f*-MWNTs (20 μg/ml) were added to the apical chamber and incubated with PBEC at 37 °C for 72 h. The radioactivity of **(A)***w*-MWNT and *w*-MWNT-ANG and **(B)***t*-MWNT and *t*-MWNT-ANG was detected in the basal chamber after crossing the PBEC monolayer. Solutions of 0.5 ml aliquots were sampled from the basal chamber at different time points. Insets to the right show the transport of *f-*MWNTs during the initial 4 h of incubation. Values are presented as mean ± S.D. (*n* = 3) with statistics analyses using One-way ANOVA test. ** *p* < 0.01; *** *p* < 0.001.

The amount of *f*-MWNTs that crossed the PBEC monolayer into the basal chamber of the Transwell™ system over a 72 h incubation period was assessed using ^111^In as a radioactive tracer. Fig. 2 shows that all studied *f*-MWNTs crossed the *in vitro* BBB model. The signals detected in the basal chamber continually increased over time, reaching 15.6 ± 1.1%, 20.3 ± 0.9%, 7.6 ± 0.3% and 11.6 ± 0.9% of the total dose for *w*-MWNT, *w*-MWNT-ANG, *t*-MWNT and *t*-MWNT-ANG, respectively, after 72 h of incubation. In general, *w*-MWNTs achieved higher percentage transport across the endothelial cells compared with *t*-MWNTs. Additionally, *w*-MWNT-ANG and *t*-MWNT-ANG showed higher percentage transport than their precursors over the studied time. Significant increases were observed by 2 h of incubation in the case of *w*-MWNTs (*p* < 0.01), whereas for *t*-MWNTs the difference became significant by 18 h (*p* < 0.001). The results indicate that conjugation of ANG to *f*-MWNTs led to a significant increase in transport across the PBEC monolayer irrespective of *f*-MWNT diameter.

The apparent permeability coefficient (P_app_) was calculated for all conjugates (Table 2). The P_app_ of *f*-MWNTs was significantly lower than that of ANG alone and the free isotope [^111^In]EDTA, which suggested that *f*-MWNTs cross the BBB at a lower rate than ANG alone. The *w*-MWNT precursor achieved higher P_app_ (0.69 ± 0.16 × 10^− 6^ cm/s) than the *t*-MWNT (0.18 ± 0.01 × 10^− 6^ cm/s) as shown in Table 2. This agreed with the higher percentage transport obtained for *w*-MWNTs by 72 h. The conjugation of ANG increased the P_app_ of *w*- and *t*-MWNT-ANG to 1.26 ± 0.09 × 10^− 6^ (54% increase) and 0.34 ± 0.02 × 10^− 6^ cm/s (67% increase) (Table 2), respectively. The studies above suggest that ANG improves *f*-MWNT targeting to the BBB *in vitro*.

### 3.6 Organ biodistribution of *f*-MWNTs in mice following i.v. administration

**Fig. 3:**
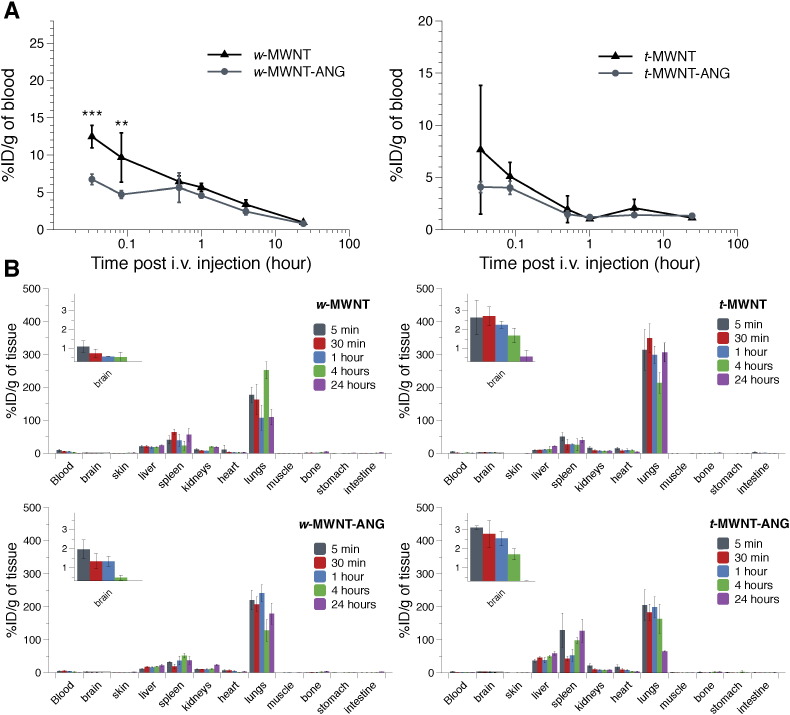
Blood and organ biodistribution profiles of radiolabelled *f*-MWNTs following i.v. administration. The blood profiles of *f*-MWNTs are shown in **(A)** and biodistribution of *f*-MWNTs in major organs is shown in **(B)**. *f*-MWNTs were injected (50 μg, 0.5 MBq) into the tail vein of C57/Bl6 mice. Blood was sampled at 5 min, 30 min, 1 h, 4 h and 24 h, and then a whole body perfusion with heparinised saline was performed before sacrifice to remove residual *f*-MWNTs from circulation. Major organs were harvested and the radioactivity of all the samples was measured using gamma counting. Insets to the left show a magnified view of the brain uptake over the studied time points. Data are presented as % injected dose per gram tissue (%ID/g). Values are expressed as mean ± S.D. (*n* = 3), and analysed using One-way ANOVA test. ** *p* < 0.01; *** *p* < 0.001.

Radiolabelled *f*-MWNTs were injected in C57/B16 mice *via* i.v. administration and the biodistribution in the major organs was monitored at specific time points using gamma counting. Fig. 3A represents blood profiles of *f*-MWNTs across the studied time points. *w*-MWNT-ANG showed a significant reduction in blood concentration compared to *w*-MWNT at the early time points (12.5 ± 1.5 vs 6.7 ± 0.7%ID/g of blood at 2 min, *p* < 0.001; 9.6 ± 3.3 vs 4.7 ± 0.5%ID/g of blood at 5 min, *p* < 0.01)). The blood circulation profiles of the two *w*-MWNTs were comparable for the rest of the time points and both decreased over time until 24 h. Unlike *w*-MWNTs, *t*-MWNT and *t*-MWNT-ANG showed a similar blood circulation profile over the studied time points (*p* > 0.05). The level in the blood also decreased over time by 24 h.

Figs. 3B and S4 show biodistribution profiles of *f*-MWNTs in the major organs. Similar organ biodistribution profiles were observed for all *f*-MWNTs conjugates with accumulation mainly in the lungs, liver and spleen at all the studied time points. The non-targeted *t*-MWNT showed higher lung accumulation than the non-targeted *w*-MWNT, agreeing with our previously published data [17].

The excretion profiles of *f*-MWNTs were examined by measuring the radioactivity in urine and faeces collected over 24 h. Fig. S5 shows that 2–5%ID/g of *f*-MWNTs were found in urine, with negligible excretion in faeces. There were no significant differences between the excretion profiles of the studied *f*-MWNTs.

### 3.7 *w*-MWNTs and *t*-MWNTs show different brain uptake profiles *in vivo*

**Fig. 4:**
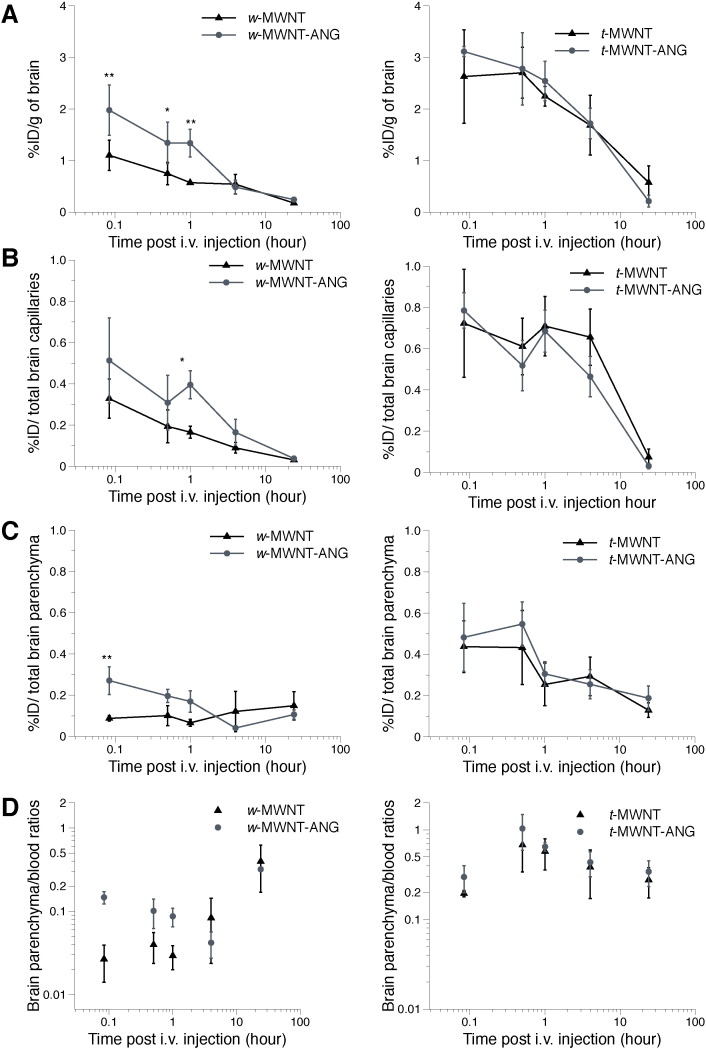
Uptake of radiolabelled *f*-MWNTs into mouse brain following i.v. administration and capillary depletion. Overall brain uptake of *f*-MWNTs is shown in **(A)** expressed as % injected dose per gram of brain (%ID/g). The radioactivity of *f*-MWNTs in brain capillaries **(B)** and parenchyma **(C)** was measured after capillary depletion with the data presented as %ID per total brain capillaries or parenchyma. Their accumulation in blood (%ID/g) versus parenchyma (%ID/g) is shown in **(D)**. The weight of parenchyma was considered as the weight of the whole brain in this case. *f*-MWNTs were injected (50 μg, 0.5 MBq) into the tail vein of C57/Bl6 mice. Whole body perfusion with heparinised saline was performed to remove residual *f*-MWNTs from circulation. The total brain accumulation was measured by gamma counting followed by capillary depletion at 5 min, 30 min, 1 h, 4 h and 24 h. Values are expressed as mean ± S.D. (*n* = 3) and analysed using One-way ANOVA test. * *p* < 0.05; ** *p* < 0.01.

The uptake of *f*-MWNTs into the whole brain, brain capillaries/parenchyma and ratios of parenchyma to blood are shown in Fig. 4 (*w*-MWNTs shown in the left panel; *t*-MWNTs in the right panel). *w*-MWNT-ANG achieved significantly higher whole brain uptake than *w*-MWNT at early time points up to 1 h post-injection (Fig. 4A left). The highest uptake was measured as 2.0 ± 0.5 and 1.1 ± 0.3%ID/g for *w*-MWNT-ANG and *w*-MWNT by 5 min post-injection, respectively. Comparable accumulation in brain for both *w*-MWNTs was measured at 4 h, followed by a reduction until 24 h post-injection. The data indicated that ANG conjugation significantly enhanced the early whole brain uptake of *w*-MWNTs.

Similar brain accumulation patterns were observed for the two *t*-MWNTs in which high uptake was measured at early time points and the values dropped gradually as a function of time (Fig. 4A right). The uptake of *t*-MWNT and *t*-MWNT-ANG reached 2.6 ± 0.9%ID/g and 3.1 ± 0.09%ID/g by 5 min post-injection, respectively. Nevertheless, *t*-MWNT-ANG did not show increased brain uptake over *t*-MWNT. It should be noted that the two *t*-MWNTs achieved notably higher brain uptake compared to non-ANG-conjugated *w*-MWNT at the studied period up to 1 h (*p* < 0.01). This was in agreement with our previous study that *t*-MWNTs appear to possess intrinsic properties that resulted in higher brain uptake than *w*-MWNTs [17]. The findings were also supported by the AUC calculations represented in Table S1.

Capillary depletion was then used to separate brain capillaries from the parenchyma. This allowed the measurement of *f*-MWNT in each respective fraction and to calculate the parenchyma to blood concentration ratio. Fig. 4B shows the amount of *f*-MWNTs in the capillary fraction. The difference of the uptake in the capillary fraction between *w*-MWNT and *w*-MWNT-ANG was significant by 1 h post-injection (0.17 ± 0.03 vs 0.4 ± 0.07%ID/capillaries, *p* < 0.05, Fig. 4B left). The amount decreased over the time course of the experiment. In the parenchyma fraction (Fig. 4C left), *w*-MWNT-ANG showed significantly greater parenchyma accumulation (0.3 ± 0.07%ID/parenchyma) than *w*-MWNT (0.08 ± 0.01%ID/parenchyma) by 5 min post-injection (*p* < 0.01).

In the case of *t*-MWNTs, there was no significant difference between the two in their uptake in either parenchyma or capillaries at all studied time points (Fig. 4B–C right). The amounts detected in the capillaries for both *t*-MWNTs were sustained up to 4 h at levels above 0.4%ID, before dropping by 24 h post-injection. *t*-MWNT and *t*-MWNT-ANG reached parenchyma with substantial amounts at 5 min post-injection (0.4 ± 0.1 and 0.5 ± 0.2%ID, respectively). In fact, both *t*-MWNTs showed significantly higher uptake in parenchyma compared to *w*-MWNTs at early time point post-injection (i.e. 5 min and 30 min, *p* < 0.01). Additionally, both *t*-MWNTs showed increased accumulation in capillaries up to 4 h post-injection compared to *w*-MWNTs (*p* < 0.05). By capillary depletion, the results overall suggested that the greater overall brain uptake for *f*-MWNTs was associated with enhanced uptake in both brain parenchyma and capillary fractions during early time points.

Differences in brain accumulation between conjugates are also expressed as parenchyma to blood ratios (P/B) calculated over the time course of the study (Fig. 4D). The values are generally used to assess the distribution of compounds in the brain parenchyma following systemic administration [26]. P/B ratios for *t*-MWNTs were generally higher than those of *w*-MWNTs at the studied period. The highest P/B ratios for the *t*-MWNT and *t*-MWNT-ANG were measured at 30 min post-injection (0.68 ± 0.34 and 1.04 ± 0.44, respectively) which were statistically higher than the values obtained for both *w*-MWNT and *w*-MWNT-ANG (*p* < 0.05). The results suggested a greater parenchyma distribution of *t*-MWNTs over *w*-MWNTs, particularly at early times post-injection. In addition, ANG brain targeting effect was only evident in the case of *w*-MWNTs.

The negative values of the elimination rate constant (Kel) obtained for *w*-MWNT and *w*-MWNT-ANG suggest better parenchymal retention of these conjugates compared to *t*-MWNTs (Table 3). Also, the Kel values of all *f*-MWNTs were higher in brain capillaries than parenchyma fractions. This observation shows that the majority of brain clearance is taking place from the capillaries while *f*-MWNTs that reached the parenchyma exhibit evidence of relative brain retention.

### 3.8 *f*-MWNTs show better accumulation in midbrain regions

**Fig. 5:**
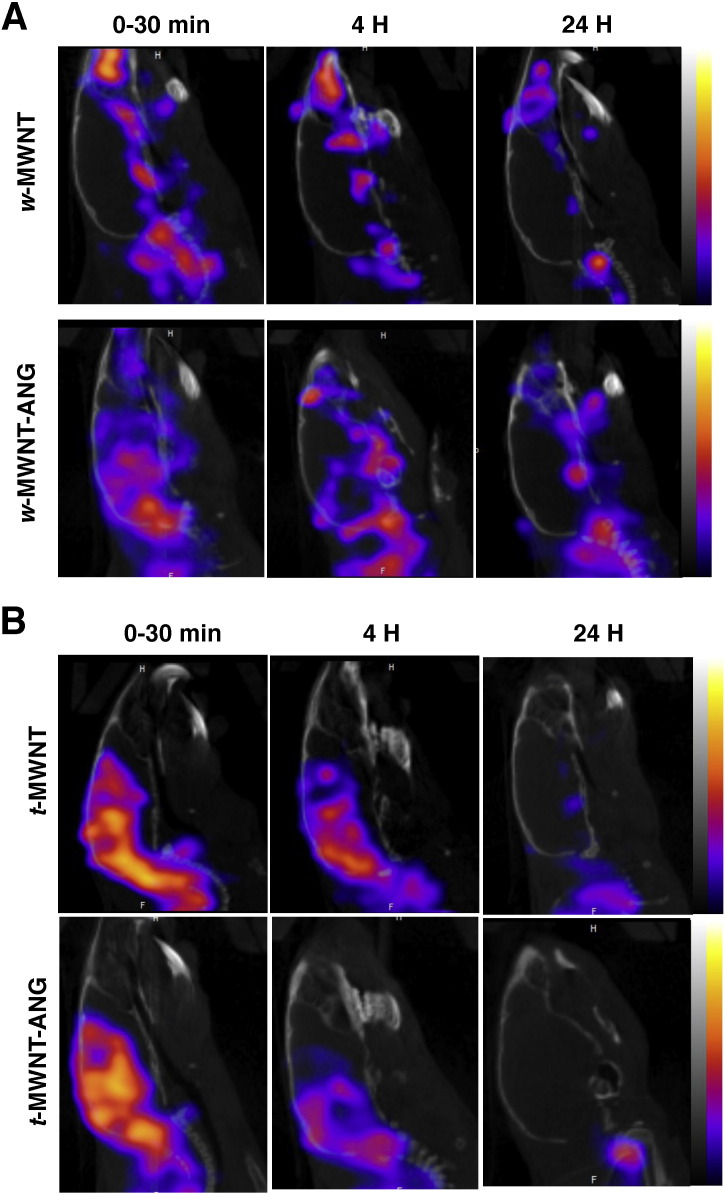
*In vivo* SPECT/CT imaging of *f*-MWNTs in mouse brain. Sagittal views of radiolabelled **(A)***w*-MWNTs and **(B)***w*-MWNTs in mouse brains at 0–30 min, 4 h and 24 h post injection. *f*-MWNTs were injected (50 μg, 5–7 MBq) into the tail vein of C57/Bl6 mice. *In vivo* 3D SPECT/CT imaging was carried out at specified time posts post i.v. injection.

The ability of radiolabelled *w*-MWNTs and *t*-MWNTs to accumulate in mouse brain after systemic administration was also studied using SPECT/CT imaging. Fig. 5A shows the brain accumulation of *w*-MWNTs. *w*-MWNT-ANG showed higher accumulation in the brain compared to *w*-MWNT by 30 min and 4 h following injection. The signals decreased after 24 h suggesting the clearance of *w*-MWNTs from brain (^111^In decay has been compensated).

Both targeted and non-targeted *t*-MWNTs achieved higher brain accumulation compared to their *w*-MWNT equivalents within 30 min of injection (Fig. 5B). Intense signals were detected in the brain for both *t*-MWNT and *t*-MWNT-ANG, with no apparent differences being detected. The intensity of the signals decreased after 4 h and diminished further after 24 h.

**Fig. 6:**
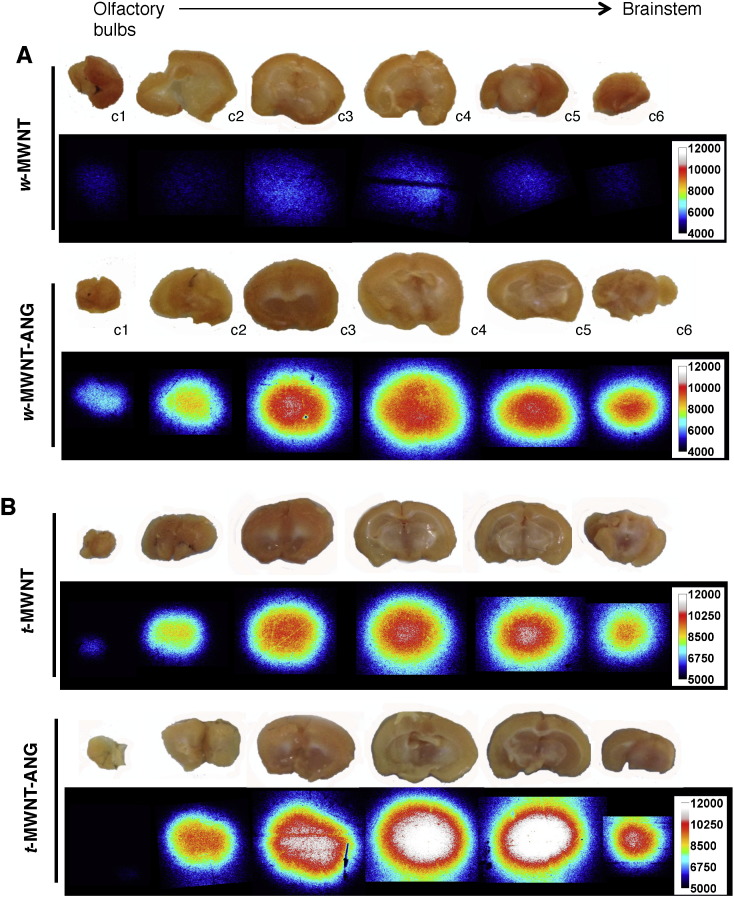
Autoradiography of *f*-MWNTs in mouse brain. Autoradiographs of brain sections from mice injected with *w*-MWNTs **(A)** and *t*-MWNTs **(B)** at 5 min post-injection. *f*-MWNTs were injected (50 μg, 5–7 MBq) into the tail vein of C57/Bl6 mice. Brains were harvested at 5 min post-injection and sectioned in coronal orientation from olfactory bulbs to brainstem into 2 mm thick sections (c1–c6).

Autoradiography provided further evidence on the region-specific brain accumulation of *w*- and *t*-MWNTs. For *w*-MWNTs, *w*-MWNT-ANG showed higher signals compared to *w*-MWNT after 5 min, matching the previous findings in SPECT/CT and gamma counting (Fig. 6A). Both *t*-MWNT and *t*-MWNT-ANG showed high autoradiography signal after 5 min (Fig. 6B). The highest signals in all studied *f*-MWNTs were localised to the thalamic region of the brain (c3–c5). It should be noted that mice were injected with *f*-MWNTs labelled with similar but not exactly the same radioactivity. Hence, direct comparison of the signals in different brain sections of the same brain (same *f*-MWNTs) can be made but not sections between different brains (different *f*-MWNTs).

### 3.9 ANG conjugation improves *w*-MWNTs uptake in glioma-bearing brains

**Fig. 7:**
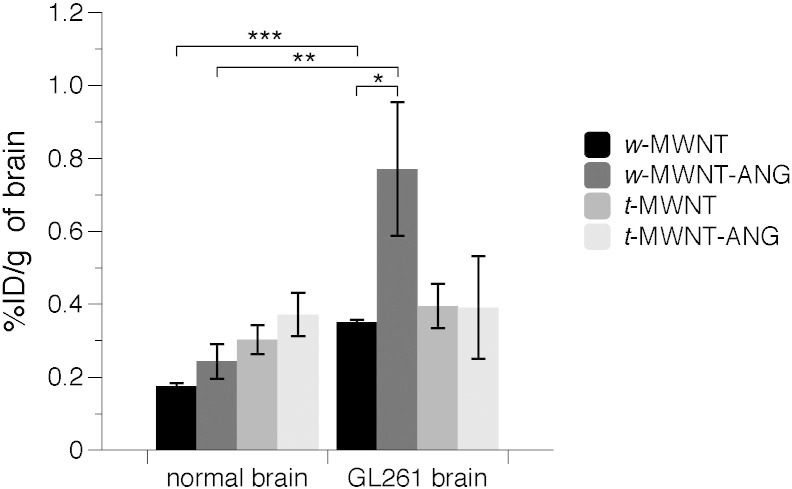
Brain uptake of *f*-MWNTs in glioma-bearing mice following i.v. administration. *w*-MWNT, *w*-MWNT-ANG, *t*-MWNT, *t*-MWNT-ANG were intravenously injected (50 μg, 0.5 MBq) into normal or GL261 glioma-bearing C57/Bl6 mice. Whole body perfusion with heparinised saline was performed to remove residual *f*-MWNTs from circulation. The total brain accumulation was measured by gamma counting at 24 h post-injection.

Finally, it was important to investigate whether the conjugation with ANG can enhance the brain uptake of *w*-MWNTs in glioma-bearing mice since LRP1 receptor is expressed on BBB and glioma [10], [11] Radiolabelled *w*-MWNTs were i.v. injected into GL261 glioma-bearing mice or normal mice and the brain uptake profiles were assessed quantitatively by γ-scintigraphy at 24 h post-injection. As shown in Fig. 7, the non-targeted *w*-MWNT displayed higher uptake in glioma brain (0.35 ± 0.01%ID/g) compared to normal brain (0.18 ± 0.01%ID/g, *p* = 0.001). Interestingly, *w*-MWNT-ANG exhibited markedly higher brain uptake in glioma brain (0.77 ± 0.17%ID/g) than normal brain (0.24 ± 0.05%ID/g, *p* < 0.01). Furthermore, a significantly enhanced uptake in glioma-brain was obtained for *w*-MWNT-ANG compared to *w*-MWNT (*p* < 0.05). No significant difference between the two *w*-MWNTs in normal brains was obtained at 24 h post-injection. Unexpectedly, no significant differences between *t*-MWNT and *t*-MWNT-ANG in normal brain or glioma brain were observed at 24 h post injection. It is not clear whether the higher elimination rate constant (Kel) values reported for *t*-MWNT than *w*-MWNT are the main reason for the lack of tumour retention, especially since measurements were taken at 24 h post-injection.

## 4 Discussion

The BBB is the main obstacle hindering the delivery of NPs to the brain using systemic administration. Several targeting ligands have been explored for NP-mediated brain targeting including transferrin [27], insulin [28], lactoferrin [29], OX26 monoclonal antibody [30] and apolipoprotein E [31]. ANG is another brain ligand which binds to LRP1, a multifunctional receptor initially known to be associated with lipoprotein metabolism, and degradation of proteases [9], [32]. The involvement of LRP1 in vasculature protection, cell migration and BBB integrity regulation was characterised later. Being engaged in several essential signalling mechanisms, LPR1 is expressed in a wide range of tissues, as well as in neurons, astrocytes [33], [34] and brain capillaries [11]. LRP1 is continually endocytosed from the membrane and recycled back [9], which makes it an attractive target for BBB crossing. Studies have shown that ANG exhibited significantly greater transcytosis capacity across bovine brain endothelial cells compared to transferrin, lactoferrin and avidin [11]. Strategies of direct conjugation of drugs to ANG have also been developed. LRP1 is abundant in many cancer cells and plays roles in oncogenesis, tumour progression and metastasis [32]; ANG has been reported to enhance brain delivery of paclitaxel, increasing the survival time of glioblastoma-bearing mice [8]. ANG has also been used for brain delivery of neurotensin to enhance analgesic response in rats [12]. Despite the success in direct coupling drug to ANG, to increase the drug payload, work has also been conducted to conjugate ANG to NPs such as polymeric micelles [13], dendrimers [14] and gold nanoparticles [15] for advanced brain delivery.

We previously reported that the diameter of *f*-MWNTs directly affects their biodistribution profiles [17]. In our recent work, we studied the behaviour of *f*-MWNTs, referred to here as *w*-MWNT, at the BBB interface. *w*-MWNT was found within brain parenchyma by 5 min following i.v. administration without the use of a targeting ligand [7]. The present work studied the effect of ANG conjugation to *f*-MWNTs of different diameters on the transport across the BBB *in vitro* and *in vivo*. The brain uptake of *w*-MWNTs and *t*-MWNTs with or without ANG conjugation was investigated in normal and glioma-bearing mice after i.v. administration. We hypothesised that targeting *f*-MWNTs to LRP1 receptor *via* ANG conjugation would offer superior BBB crossing and therefore brain accumulation compared to non-targeted *f*-MWNTs. Furthermore, ANG-conjugated *f*-MWNTs would favour glioma tumours over healthy brain tissues *in vivo*.

Both *w*- and *t*-MWNT-ANG clearly showed significant increases in the percentage transport across PBEC compared to *w*- and *t*-MWNT, indicating the ANG targeting effect *in vitro*. Also, interestingly, regardless of ANG conjugation, *w*-MWNTs showed superior percentage transport across the *in vitro* BBB model compared to *t*-MWNTs. The enhanced uptake of *w*-MWNT-ANG over *w*-MWNT across PBEC was observed after 2 h of incubation while the targeting effect took place after longer incubation time (i.e. 18 h) in the case of *t*-MWNTs.

Based on our previous report elucidating the mechanisms of *f*-MWNTs transport across the *in vitro* BBB model [7], it is unlikely that *f*-MWNTs enter the cells *via* a receptor-mediated mechanism as the dimension is too large for this route (> 500 nm in length) to be utilised. However, coupling ANG to *f*-MWNTs may possibly provide anchoring points to the LRP1 receptor on the cell surface and thereby increase the cellular uptake of targeted *w*-MWNT-ANG and *t*-MWNT-ANG.

*In vivo* results showed that *w*-MWNT-ANG exhibited a significant improvement over *w*-MWNT in overall brain uptake up to 1 h post-injection. Capillary depletion revealed that the enhancement in whole brain uptake for *w*-MWNT-ANG at earlier time points was due to the higher accumulation in the brain parenchyma and capillaries compared to non-targeted *w*-MWNT.

By contrast, no significant difference was observed between the two *t*-MWNTs in their accumulation in the whole brain, capillaries or parenchyma. There are several possible reasons for the lack of targeting effect of *t*-MWNT-ANG *in vivo*. Both *t*-MWNTs may have reached a level of saturation in brain capillaries right after injection. As shown in Fig. 4B, significantly higher %ID/capillaries was measured for both *t*-MWNTs compared to non-targeted *w*-MWNT up to 4 h post-injection. The targeting effect of ANG might be surpassed by the higher intrinsic ability of *t*-MWNTs to cross the BBB. It is also worth mentioning that the amount of ANG loading on *t*-MWNT-ANG was ~ 4 fold lower than for *w*-MWNT-ANG which might have been insufficient to enhance the brain uptake *in vivo*.

In addition to the uptake enhancement imparted by ANG conjugation, *f*-MWNTs themselves play a major role in determining the extent of uptake in brain. Studies in a non-BBB environment have shown that rod-shaped nanoparticles achieved greater uptake into a human cervical carcinoma cell line compared to the spherical NPs due to the difference in aspect ratios [35], [36]. Accordingly, the tubular-shaped *f*-MWNTs and their high aspect ratios (~ 58 and ~ 30 for *t*-MWNTs and *w*-MWNT, respectively) might explain their ability to reach the brain parenchyma after i.v. injection in quantities higher than the values reported for other NPs of lower aspect ratios.

Even without active targeting, a maximum *in vivo* brain uptake for non-targeted *w*- and *t*-MWNTs was 1.1 ± 0.3 and 2.4 ± 0.9%ID/g of brain, respectively. The values were several fold higher than those reported for non-targeted liposomes ~ 0.138 ± 0.03%ID/g [37], ANG-targeted polymeric micelles ~ 0.6%ID/g [13], ANG-modified dendrimers ~ 0.25%ID/g [14], [27] and non-targeted polymersomes ~ 0.3%ID/g [38]. The highest brain accumulation in our study was measured by 5 min post-injection (with perfusion in place) compared with other systems which generally showed the highest brain uptake between 1 and 4 h post-injection.

As mentioned previously, ANG can be utilised as a ‘doubled-targeting’ ligand, to target not only the BBB but also brain tumours. Many studies have developed ANG-tailored NPs as nanocarriers to achieve efficient drug delivery to brain tumours. It has been reported that ANG-conjugated cationic liposomes successfully co-delivered therapeutic DNA and paclitaxel to glioma *in vivo*, resulting in longer survival time than controls and even commercial temozolomide treatments. Ren et al. have investigated the effect of ANG on *f*-MWNT in which *f*-MWNT was coated with ANG-conjugated polyethylene glycol (PEG) and also incorporated doxorubicin (Dox). Optical imaging showed superior brain accumulation and prolonged survival time for Dox-MWNT-PEG-ANG compared to Dox-MWNT-PEG or Dox alone in glioma-bearing mice [39]. ANG-modified gold nanoparticles have been shown to significantly enhance tumour accumulation of Dox in glioma-bearing mice and increased survival time compared to non-targeted NPs or Dox alone [15]. It is worth mentioning that none of the above studies provide quantification of NPs in glioma brain but relied on semi-quantitative fluorescence imaging of doxorubicin or fluorescent liposomes. By contrast, the present study quantified the brain accumulation of the nanocarriers directly.

*f*-MWNTs reached brain rapidly, with high brain uptake detected right after injection then decreasing as a function of time. An altered brain uptake pattern was observed for *w*-MWNTs in the glioma model. At 24 h after injection, both *w*-MWNTs exhibited higher brain uptake in glioma-bearing mice than normal mice. The slight increase of *w*-MWNT lacking ANG in glioma brain could be due to a relatively disrupted BBB [40]. ANG-mediated targeting and/or retention in glioma brain could account for the substantial amount of *w*-MWNT-ANG (~ 0.8%ID/g) measured at 24 h post-injection compared to non-targeted *w*-MWNT (~ 0.38%ID/g). *t*-MWNTs, on the other hand, showed lower glioma accumulation than *w*-MWNTs after 24 h. This could be due to the thinner diameter of *t*-MWNTs that led to faster clearance rate from the brain glioma. Further, studies of time-course in combination with imaging are required to understand how ANG influences glioma targeting of *f*-MWNTs.

## 5 Conclusions

ANG conjugation to both *w-* and *t-*MWNT significantly enhanced their transport across the PBEC monolayer *in vitro*. However, *in vivo*, ANG conjugation significantly increased brain uptake of *w*-MWNT but not *t*-MWNT. The latter, however, exhibited intrinsically higher brain uptake, with and without ANG conjugation. Furthermore, *w*-MWNT-ANG exhibited enhanced uptake in glioma brain compared to non-targeted conjugate, supporting the use of ANG-conjugated *f*-MWNTs for effective drug delivery to brain tumours.

## Figures and Tables

**Table 1:** Functionalised *f*-MWNTs characteristics.

*f*-MWNTs	*f*-MWNTs code	Mean diameter^a^ (nm) ± S.D.	Mean length^a^ (nm) ± S.D.	Total functionalisation degree (amidation + cycloaddition)^b^ (μmol/g CNTs)	DTPA loading^b^ (μmol/g CNTs)	Angiopep-2 loading^b^ (μmol/g CNTs)
MWNT **4**	*w*-MWNT	23.8 ± 2.1	494 ± 54	262 (0 + 262)	234	–
MWNT **5**	–	23.8 ± 2.1	494 ± 54	262 (0 + 262)	234	–
MWNT **6**	*w*-MWNT-ANG	23.8 ± 2.1	494 ± 54	262 (0 + 262)	234	31.2
MWNT **12**	*t*-MWNT	7.9 ± 1.8	459 ± 61	837 (467 + 97)	71	–
MWNT **13**	–	7.9 ± 1.8	459 ± 61	837 (467 + 97)	71	–
MWNT **14**	*t*-MWNT-ANG	7.9 ± 1.8	459 ± 61	837 (467 + 97)	71	7.6

a Analysed by TEM measurements of *f*-MWNTs **4** and **13**, for *w*-MWNTs and *t*-MWNTs, respectively. (*n* = 50).

b Analysed by TGA.

**Table 2:** Apparent permeability coefficient (P_app_) of *f*-MWNTs across PBEC.

	*w*-MWNT	*w*-MWNTs-ANG	*t*-MWNT	*t*-MWNT-ANG	Angiopep-2	[^111^In]EDTA
P_app_ (cm/s) × 10^− 6^	0.69 ± 0.16	1.26 ± 0.09	0.18 ± 0.01	0.34 ± 0.02	8.69 ± 1.53	1.03 ± 0.06

**Table 3:** Elimination rate constant (Kel) of *f*-MWNTs in brain.

MWNT type	Whole brain (hour^− 1^)^a^	Capillaries (hour^− 1^)^a^	Parenchyma (hour^− 1^)^a^
w-MWNT	0.053 ± 0.008	0.066 ± 0.016	− 0.026 ± − 0.013
w-MWNT-ANG	0.060 ± 0.013	0.092 ± 0.026	− 0.005 ± − 0.001
t-MWNT	0.058 ± 0.013	0.102 ± 0.032	0.034 ± 0.011
t-MWNT-ANG	0.107 ± 0.031	0.135 ± 0.035	0.019 ± 0.005

a The data expressed as mean ± SD, *n* = 3.
