# Supplementary material for: Translocation of LRP1 targeted carbon nanotubes of different diameters across the blood–brain barrier in vitro and in vivo
Source: J Control Release. 2016 Mar 10;225:217–29. doi: 10.1016/j.jconrel.2016.01.031 (PMC4778610; doi:10.1016/j.jconrel.2016.01.031)
Supplement: Supplementary file 1 — Supplementary material. [file mmc1.docx]

**Translocation of LRP-1 targeted carbon nanotubes of different diameters across the blood-brain barrier in vitro and in vivo**

Houmam Kafa^1†^, Julie Tzu-Wen Wang^1†^, Noelia Rubio^1^, Rebecca Klippstein^1^, Pedro M Costa^1^, Hatem AFM Hasan^1^, Jane K Sosabowski^2^, Sukhvinder S Bansal^1^, Jane E Preston^1^, N Joan Abbott^1^ and Khuloud T Al-Jamal^1^*

^1^ Institute of Pharmaceutical Science, Faculty of Life Sciences & Medicine, King's College London, Franklin-Wilkins Building, 150 Stamford Street, London SE1 9NH, UK

^2^ Centre for Molecular Oncology, Barts Cancer Institute, Queen Mary University of London, London EC1M 6BQ, UK

^†^ These authors contributed equally to this work

Address correspondence to:

* Dr Khuloud T. Al-Jamal

Institute of Pharmaceutical Science

King's College London

Franklin-Wilkins Building

150 Stamford Street

London SE1 9NH, UK

E-mail: [khuloud.al-jamal@kcl.ac.uk](mailto:khuloud.al-jamal@kcl.ac.uk)

***SI Text***

***Materials***

Chemicals for the MWNTs synthesis, sodium chloride, trypsin solution from porcine pancreas, cytosine arabinoside, poly-*L*-lysine, Hanks’ Balanced Salt solution (HBSS), paraformaldehyde, HEPES, N,N-dimethylformamide, 8-(4-Chlorophenylthio)adenosine 3',5'-cyclic monophosphate sodium salt, 4-(3-butoxy-4-methoxybenzyl)imidazolidin-2-one, hydrocortisone, Dounce tissue grinder set, fibronectin from bovine plasma, N,N-diisopropylethylamine, diethylenetriaminepentaacetic acid, dimethyl sulfoxide, heparin sodium salt from porcine intestinal mucosa, puromycin dihydrochloride from *Streptomyces alboniger*, Triton™ X-100 and Dextran from *Leuconostoc spp* were all obtained from Sigma-Aldrich, UK. Silica gel impregnated glass fibre sheets for thin layer chromatography (TLC) were purchased from Agilent Technologies, UK. Precision bottle-top filter unit MF75 series disposable SFCA membrane and 70 µm cell strainer were obtained from BD Falcon™, UK. Newborn calf heat-inactivated serum and bovine plasma-derived serum were obtained from First-Link, UK Ltd. High glucose Dulbecco’s modified Eagle’s Medium (DMEM), Low glucose DMEM, medium 199, Advanced RPMI, phosphate buffered saline (PBS), 10x, pH 7.4, minimum essential medium/HEPES, penicillin-streptomycin 100X, 0.05% Trypsin-EDTA (1X) with phenol red and GlutaMAX™ supplement were obtained from Invitrogen, Life Technologies, UK. Collagenase type 3, trypsin powder, deoxyribonuclease I were obtained from Worthington Biochemicals Inc, USA. Nylon mesh 60 μm and 150 μm pore size, 50 mm disks were purchased from Plastok, UK. Ethylenediaminetetraacetic acid (EDTA) was purchased from Alfa Aesar, UK. Tris(2-carboxyethyl)phosphine hydrochloride (TCEP) was purchased from ThermoFisher, UK.

***ANG synthesis by solid phase peptide synthesis (SPPS)***

A modified ANG sequence (TFFYGGSRGKRNNFKTEEYG) [[1](#_ENREF_1)] was synthesised using the CEM Liberty 1 microwave peptide synthesizer (CEM Microwave Technology Ltd, UK) on NovaSyn® TGR resin (0.5 mmol/g loading). Lys^15^ (K^15^) was protected with a Dde group. The Dde group was selectively removed with hydrazine hydrate while the peptide was attached to the resin. The Lys^15^ ε-amino group was subsequently acylated with a cysteine. Propargylglycine (G20) was conjugated to facilitate fluorescence labeling when required. Fmoc groups were deprotected with 20% piperidine in dimethylformamide (DMF) v/v (10 min x 2). Acylation cycles were carried out by mixing each of the amino acids with O-(6-Chlorobenzotriazol-1-yl)-N,N,N′,N′-tetramethyluronium hexafluorophosphate (HCTU) (0.78 mmol) in the presence of 2,3,5-Collidine (1.6 mmol) in 3 ml of pure DMF. The mixture was incubated with the solid resin for 10 min using a microwave power of 25 W. The Dde group on K15 was selectively de-protected using a solution of hydrazine hydrate in DMF (2% v/v). The free amino group on K15 was acylated with an Fmoc-cys(Trt)-OH residue (70 mg, 119 µmol) in 3 ml of DMF for 1 hour in RT. The synthesised peptide was cleaved from the solid resin and simultaneously deprotected using 4 ml of trifluoroacetic acid (TFA), water, phenol, triisopropylsilane, thioanisole and 2,2′(Ethylenedioxy)diethanethiol (77.5/5/5/5/5/2.5) for 3 hours. The cleaved peptide was precipitated in cold diethyl ether and washed three times with centrifugation (3220 g). The solid pellet was dissolved in deionised water and lyophilised. The molecular weight of the modified ANG was confirmed by MALDI-TOF MS (Bruker, USA) using α-cyano-4-hydroxycinnamic acid as the matrix. The modified ANG was also characterised by reverse phase high-performance liquid chromatography (RP-HPLC) using Vydac 218TP54 column (manufacture, USA) with a water/acetonitrile gradient mobile phase containing 0.1% TFA. The eluent was detected using a UV-vis detector at 280 nm.

***Preparation of functionalised w-MWNT and conjugation with Angiopep-2***

Synthetic steps are described in **Scheme 1**. Pristine MWNT (100 mg), with a diameter of 20-30 nm, were suspended in 100 ml of dimethylformamide (DMF) and sonicated for 10 min. After sonication, tertiary-butyloxycarbonyl (Boc)-protected amino acid (187.5 mg, 0.68 mmol), phthalimide-protected amino acid (208.4 mg, 0.68 mmol) and paraformaldehyde (40.1 mg, 1.36 mmol) were added stepwise (79.2 mg amino acids and 8 mg paraformaldehyde every 24 hours), and the mixture was heated at 125 ^◦^C for 5 days. Multiple centrifugation steps (1811 g, 10 min) in DMF were carried out to separate the unreacted MWNT (remain suspended) from the functionalised *w*-MWNT, which were then filtered through a 0.2 μm PTFE filter and the collected black solid was washed with 100 ml of dimethylformamide (DMF) and methanol yielding 41.7 mg of **1** (7.6 % mass loss at 600 ^◦^C by TGA corresponding to 137.7 µmol and 124 µmol of Boc- and phthalimide-protected amino acid/g CNTs, respectively).

### MWNT 2

To eliminate the Boc protecting group, 40 mg of **1** were suspended in 10 ml dichloromethane (DCM), and sonicated for 10 min. After sonication, 10 ml of trifluoroacetic acid (TFA) were added to the reaction mixture. The solution was stirred for 24 hours and the *w*-MWNT were filtered through a 0.2 μm PTFE filter. The collected black solid was washed with 100 ml of DMF and methanol yielding 30 mg of **2** (0.7 % mass loss at 600 ^◦^C by TGA corresponding to 69.3 µmol Boc/g CNTs).

### MWNT 3

For the preparation of DTPA-MWNT, 30 mg of derivative **2** were dispersed in 30 ml of dry DMF, and the ammonium groups were neutralised with diisopropylethylamine (3.7 µl, 27 µmol at 3:1 excess molar ratio). Diethylene triamine pentaacetic acid (DTPA) was added to **2** (3.2 mg, 90 µmol at 1:1 molar ratio), and the reaction was stirred for 48 hours at 60 ^◦^C under nitrogen atmosphere to avoid the hydrolysis of the anhydride groups in the presence of water in air. The mixture was filtered through a 0.2 μm PTFE filter, re-suspended in 100 ml DMF and sonicated for 10 min. Several washing steps were then carried out on the sample in methanol to remove unbound DTPA from the mixture. The filtrate was re-suspended in 10 ml of methanol and dialysed against deionised water (MW cut off size: 10,000 Dalton) for 48 hours in water. Finally, the mixture was filtered yielding 20 mg. Derivative **3** was characterised by TGA (8.8 % mass loss at 600 ^◦^C by TGA corresponding to 233.8 µmol DTPA/g CNTs).

### MWNT 4

To eliminate the phthalimide protecting group, 40 mg of derivative **3** were suspended in 20 ml DMF, and sonicated for 10 min. After sonication, hydrazine hydrate (5.4 ml, 165 µmol) were added to the reaction mixture. The solution was stirred for 24 hours and the *w*-MWNTs were filtered through a 0.2 μm PTFE filter. The collected black solid was washed with 100 ml of DMF and methanol yielding 16.2 mg of derivative **4** (4.3 % mass loss at 600 ^◦^C by TGA corresponding to 294.3 µmol functional groups/g CNTs).

### MWNT 5

For the purpose of conjugating Angiopep-2 peptide to the MWNT, the amine functional groups in derivative **4** were modified with maleimide. MWNT **4** were suspended in 10 ml DMF and sonicated for 10 min. The ammonium groups were neutralised with diisopropylethylamine (17.4 µl, 100 µmol at 50:1 excess molar ratio). 3-maleimidopropionic acid N-hydroxysuccinimide ester (BMPS) was then added (10.7 mg, 40 µmol at 20:1 excess molar ratio), and the reaction was stirred for 24 hours at room temperature under nitrogen atmosphere. The mixture was filtered through a 0.2 μm PTFE filter, and washed twice in 100 ml methanol. The mixture was filtered and the black solid was collected yielding 6.2 mg. Derivative **5** was characterised by TGA (10.4% mass loss at 600 ^◦^C by TGA corresponding to 684.2 µmol of maleimide/g CNTs).

***Preparation of functionalised w-MWNT and conjugation with Angiopep-2***

**MWNT 7**

Synthetic steps are described in **Scheme 2**. Pristine *t*-MWNTs (300 mg), with a diameter of 7.8±1.5 nm, were suspended in a mixture sulphuric acid:nitric acid (3:1) (75 mL). The suspension was sonicated for 12 hours. 500 ml of DI water was then added to the CNT suspension and the mixture was filtered through a 0.4 µm GTTP filter. The collected black solid was washed with DI water (300 mL), 5 % NaOH solution (300 mL) and methanol (200 mL) yielding 250 mg of **7** (15.9 % mass loss at 600 ^◦^C by TGA corresponding to 3.3 mmol carboxylic groups/g CNTs).

### MWNT 8

MWNT **7** was further functionalised using the 1, 3-dipolar cycloaddition reaction. 100 mg of MWNT **7** were suspended in 100 ml of DMF and sonicated for 10 min. After sonication, the tertiary-butyloxycarbonyl (Boc)-protected amino acid (350 mg, 1.3 mmol) and paraformaldehyde (39 mg, 1.3 mmol) were added stepwise (70 mg amino acid and 7.8 mg aldehyde every 24 hours), and the mixture was heated at 125 ^◦^C for 5 days. Multiple centrifugation steps (1811 g, 10 min) in DMF were carried out to separate the unreacted MWNTs (remain suspended) from the functionalised *t*-MWNT, which were then filtered through a 0.2 μm PTFE filter and the collected black solid was washed with 100 ml of dimethylformamide (DMF) and methanol yielding 42 mg of **8** (12.9 % mass loss at 600 ^◦^C by TGA corresponding to 467.4 µmol Boc-protected amino acid/g CNTs).

### MWNT 9

MWNT **8** were further functionalised using the amidation reaction. 40 mg of **9** were suspended in 40 mL of DMF and sonicated for 10 min. After sonication, 1-ethyl-3-(3-dimethylaminopropyl)carbodiimide hydrochloride (EDC·HCl) (17 mg, 0.09 mmol) and *N*-hydroxybenzotriazole (12 mg, 0.09 mmol) were added. Phthalamide-protected amino acid (16 mg, 0.06 mmol) was then added to the reaction mixture and was stirred for 48 hours. The reaction mixture was then filtered through a 0.2 μm PTFE filter and the collected black solid was washed with 100 ml of dimethylformamide (DMF) and methanol yielding 38 mg of **9**. (5.5 % mass loss at 600 ^◦^C by TGA corresponding to 96.9 µmol diamine linker/g CNTs).

### MWNT 10

For the deprotection of phthalimide protecting group, 38 mg of MWNT **9** were suspended in 30 mL of DMF and sonicated for 10 min. Hydrazine monohydrate (13.5 mL 417.4 µmol) was added to the reaction mixture and the suspension was stirred for 24 h. The suspension was then filtered through a 0.2 μm PTFE filter and the collected black solid was washed with 100 ml of dimethylformamide (DMF) and methanol yielding 37 mg of **10** (13.6 % mass loss at 600 ^◦^C by TGA corresponding to 930 µmol phthalimide/g CNTs).

### MWNT 11

36 mg of **10** were dispersed in 40 mL of dry DMF, and the ammonium groups were neutralised with diisopropylethylamine (6.1 µl, 43.2 µmol at 3:1 excess molar ratio). Diethylene triamine pentaacetic acid (DTPA) was added to the *t*-MWNT (5.14 mg, 14.4 µmol at 1:1 excess molar ratio), and the reaction was stirred for 48 hours at 60 ^◦^C under nitrogen atmosphere to avoid the hydrolysis of the anhydride groups in the presence of water in air. The mixture was filtered through a 0.2 μm PTFE filter, re-suspended in 100 ml DMF and sonicated for 10 min. Several washing steps were then carried out on the sample in methanol to remove unbound DTPA from the mixture. The filtrate was re-suspended in 10 ml of methanol and dialysed against deionised water (MW cut off size: 10,000 Dalton) for 48 hours in water. Finally, the mixture was filtered yielding 33.2 mg. Derivative **11** was characterised by TGA (6.3 % mass loss at 600 ^◦^C by TGA corresponding to 167.4 µmol DTPA/g CNTs).

### MWNT 12

For the deprotection of Boc group, 30 mg of **11** were suspended in 15 ml DMF, and sonicated for 10 min. After sonication, 15 ml of trifluoroacetic acid (TFA) were added to the reaction mixture. The solution was stirred for 24 hours and the *t*-MWNTs were filtered through a 0.2 μm PTFE filter. The collected black solid was washed with 100 mL of DMF and methanol yielding 26.6 mg of **12** (2.8 % mass loss at 600 ^◦^C by TGA corresponding to 277.2 µmol Boc/g CNTs).

### MWNT 13

For the purpose of conjugating Angiopep-2 peptide to the *t*-MWNT, the amine functional groups in MWNT **12** were modified with maleimide. 12 mg of MWNT **12** were suspended in 15 ml DMF and sonicated for 10 min. the ammonium groups were neutralised with diisopropylethylamine (24 µl, 166.2 µmol at 50:1 excess molar ratio). BMPS was then added (17.7 mg, 66.4 µmol at 20:1 excess molar ratio), and the reaction was stirred for 48 hours at room temperature under nitrogen atmosphere. The mixture was filtered through a 0.2 μm PTFE filter, and washed twice in 100 ml methanol. The mixture was filtered and the black solid collected yielding 6.2 mg. Derivative **13** was characterised by TGA (4.4 % mass loss at 600 ^◦^C by TGA corresponding to 289.5 µmol functional groups/g CNTs).

### *Characterisation of f-MWNTs derivatives by* *thermogravimetric analysis (TGA)*

*f*-MWNTs samples (0.4 to 1.2 mg) were loaded in platinum pans and the TGA analysis was performed using the TGA Q500 (TA instruments, USA). The analysis was carried out isothermally in nitrogen atmosphere at 100 ˚C for 20 min followed by a controlled increase in temperature at a rate of 10 ˚C/min reaching a maximum of 1000 ˚C at the end of the analysis.

###

### *Transmission Electron Microscopy*

*f*-MWNTs samples were dispersed in water (1 mg/ml). A few drops of each sample were deposited on carbon/formvar-coated 300-mesh copper grids, and then allowed to dry on filter paper. The grids were imaged on a Philips CM12 transmission electron microscope at 80 kV acceleration voltage, with 150 µm objective aperture. Length and diameter measurements were performed using NIH ImageJ software (*n*=50).

**Reference**

[1] Mei L, Zhang Q, Yang Y, He Q, Gao H. Angiopep-2 and activatable cell penetrating peptide dual modified nanoparticles for enhanced tumor targeting and penetrating. Int J Pharm. 2014;474:95-102.

***SI Tables***

**Table S1.** Area under the curve analysis

^a^ The data expressed as mean±SD, *n*=3

|  | ***f*-MWNT type** | **Whole brain (%ID.g^-1^.h)^a^** | **Capillaries**  **(%ID.g^-1^.h) ^a^** | **Parenchyma**  **(%ID.g^-1^.h) ^a^** |
| --- | --- | --- | --- | --- |
| **1 hour** | ***w*-MWNT** | 0.75±0.15 | 0.21±0.06 | 0.08±0.02 |
|  | ***w*-MWNT-ANG** | 1.44±0.36 | 0.36±0.12 | 0.20±0.05 |
|  | ***t*-MWNT** | 2.45±0.50 | 0.64±0.17 | 0.37±0.14 |
|  | ***t*-MWNT-ANG** | 2.69±0.39 | 0.60±0.10 | 0.44±0.11 |
| **24 hour** | ***w*-MWNT** | 8.91±1.75 | 1.68±0.48 | 3.05±1.31 |
|  | ***w*-MWNT-ANG** | 10.94±2.66 | 2.87±0.88 | 1.84±0.42 |
|  | ***t*-MWNT** | 28.92±8.82 | 8.02±2.43 | 5.19±1.77 |
|  | ***t*-MWNT-ANG** | 23.40±5.39 | 5.50±1.23 | 5.67±1.51 |

***SI Figures***

**
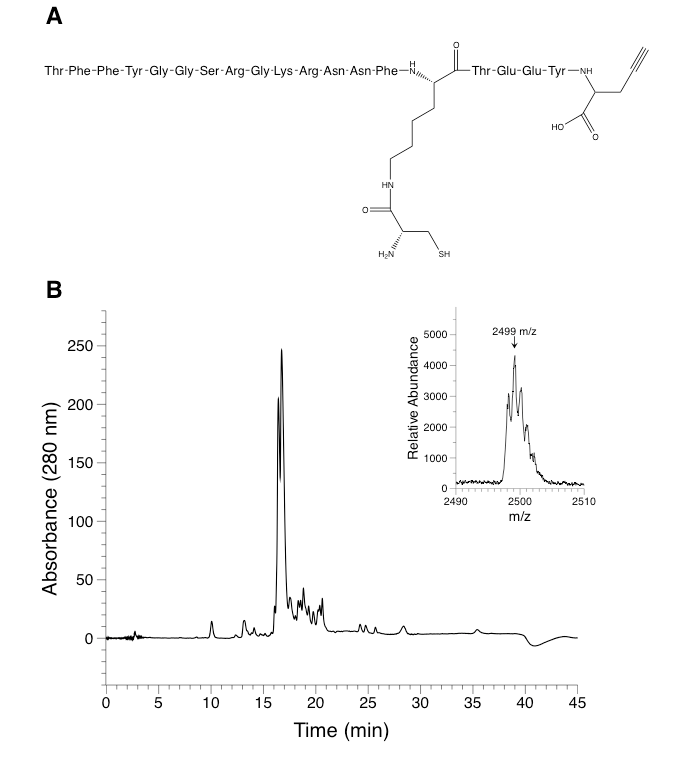
**

**Figure S1: Structure and chromatographic analysis of Angiopep-2 using HPLC and mass spectrometry**. SPPS was used for Angiopep-2 synthesis. **(A)** The structure of Angiopep-2 (TFFYGGSRGKRNNFKTEEYG) contained propargylglycine at the C-terminal. Cysteine residue was post-conjugated to the lysine at position 15. **(B)** HPLC analysis was performed using a Vydac 218TP54 stationary phase and a gradient mobile phase (A: 0.1% TFA, B: 90% acetonitrile) with a flow rate equals to 1 ml/min. The UV detection was measured at 280 nm. A single peak in the HPLC chromatogram (retention time: 16.7 min) confirmed the purity of the synthesised Angiopep-2. Inset on the right is MALDI-TOF MS using cyano-4-hydroxycinnamic acid as the matrix. The highest abundant ion was 2499 m/z corresponding to the Angiopep-2 molecular weight.

**Figure S2: Thermogravimetric analysis of *f*-MWNTs under N_2_ atmosphere**. The mass loss observed at 600 ˚C was attributed to the presence of organic functional groups with lower decomposition temperature than pristine MWNT. **(A)** TGA analysis of wide MWNT. MWNT **4** showed mass loss following the functionalisation reactions. This weight loss further increased in MWNT **5** following the conjugation of the maleimide group. The mass observed with MWNT **6** was due to the conjugation of Angiopep-2 peptide. **(B)** TGA analysis of thin MWNT. The mass loss associated with MWNTs **12** was due to the introduction of functional groups in the functionalisation reactions. The weight loss further increased after the conjugation of maleimide and Angiopep-2 to MWNT **13** and MWNT **14**, respectively. The µmoles of functional groups were calculated from the percentage mass loss by knowing the molecular weight of the side chains. Degrees of functionalisation are presented in **Table 1**.

**Figure S3: Radiolabelling studies of *f*-MWNTs with ^111^Indium.** Chromatograms showing radiolabelling efficiency of *w*-MWNTs **(A)** and *t*-MWNTs **(B)** after the labelling reaction with ^111^In, and prior to injection**.** TLCs were generated using 0.1 M ammonium acetate buffer containing 50 mM EDTA. Radiolabelled *f*-MWNTs appear as an immobile spot at the application point while [^111^In]EDTA appears as a mobile spot at the solvent front, representing the un-labelled fraction. **(C)** Labelling stability of the radiolabelled *f*-MWNTs in 50 % serum at 37 °C for 24 hours.

**Figure S4: Organ biodistribution of radiolabelled *f*-MWNTs following i.v. administration.** *f*-MWNTs were injected (50 µg, 0.5 MBq) into the tail vein of C57Bl6 mice. The blood was sampled at 5 min, 30 min, 1 h, 4 h and 24 hours, and a whole body perfusion with heparinised saline was performed before sacrifice to remove residual *f*-MWNTs from circulation. All the major organs were harvested and the the radioactivity was measured using gamma counting. Highest signals were measured in the lungs, liver and spleen. Insets to the left show a magnified view of the brain uptake over the studied time points. Data is presented as % injected dose per organ (% ID/organ). Values are expressed as mean ± S.D. (*n=*3).

**Figure S5: Excretion profile of *f*-MWNTs following i.v. administration in mice.** *f*-MWNTs were injected (50 µg, 0.5 MBq) into the tail vein of C57Bl6 mice. The mice were starved for 24 hours and urine and faeces were collected in metabolic cages. The signal in the collected samples were measured using gamma counting. The majority of excreted *f*-MWNTs were found in urine, and no significant differences between the precursor and targeted *f*-MWNTs were found. Data is presented as % injected dose (% ID). Values are expressed as mean ± S.D. (*n=*3).
